# EHMTI-0045. The succession of aura and headache: a prospective diary-based study

**DOI:** 10.1186/1129-2377-15-S1-D73

**Published:** 2014-09-18

**Authors:** M Viana, M Linde, G Sances, N Ghiotto, E Guaschino, M Allena, G Nappi, PJ Goadsby, C Tassorelli

**Affiliations:** 1 Headache Science Center, C. Mondino National Neurological Institute, Pavia, Italy; 2 Norwegian National Headache Centre Department of Neuroscience, Norwegian University of Science and Technology, Trondheim, Norway; 3 Headache Group – NIHR-Wellcome Trust Clinical Research Facility, King's College London, London, UK

## Introduction

Evaluation of clinical characteristic of migraine with aura, such as timing of succession between aura and headache, is important as it might give us an insight in migraine with aura pathophysiology. At the best of our knowledge no study assessed with a prospective diary specifically the time of onset/end of aura and headache. Few prospective studies inquired generally about the presence of one symptom (aura or headache) during the other one (Hansen et al 2012).

## Aims

To evaluate the temporal relationship between aura onset/end and headache with a prospective diary-based study.

## Methods

We recruited 136 consecutive patients affected by non-hemiplegic migraine aura at the Headache Centers of Pavia and Trondheim. All the patients prospectively recorded the characteristics of three consecutive attacks in an ad hoc aura diary that included the time of onset and the end of each aura symptoms and headache.

## Results

Of the 136 patients recruited so far, 44 completed the diaries during three consecutive auras for a cumulative number of 132 auras recorded. In 14 attacks, headache (HA) did not follow aura, and in 26 attacks HA was present but we do not have any information about its onset. Of the remaining 92 auras, in 9 (10%) HA started before aura, in 10 (11%) HA started simultaneously with aura; in 28 (30%) HA started during aura, in 13 (14%) auras HA started when aura stopped, in 32 (35%) HA started after a free interval of time after the end of aura (see Figure).

## Conclusions

The headache phase of migraine with aura may either start before, simultaneously, during or after, the end of aura.

No conflict of interest.

